# Structural data of lanthanide complex constructed by 4-iodo-3-methyl benzoic acid and 4,7-dimethyl-1,10-phenanthroline

**DOI:** 10.1016/j.dib.2018.09.063

**Published:** 2018-09-27

**Authors:** Yongli Zhao, Ting Tang, Qingrong Yang, Ziqi Liu

**Affiliations:** College of Chemistry and Chemical Engineering, Key Laboratory of Functional Small Organic Molecule, Ministry of Education and Jiangxi׳s Key Laboratory of Green Chemistry, Jiangxi Normal University, Nanchang 330022, PR China

## Abstract

In this data article, we present the FT-IR and PXRD data of the lanthanide complexes constructed by 4-iodo-3-methylbenzoic acid (IMBA) and 4,7-dimethyl-1,10-phenanthroline (dmp). Detailed structure analysis, luminescence and sensing properties were discussed in our previous study, “Highly Luminescent Lanthanide Complexes as Bifunctional Sensor for Et_2_O and Fe^2+^” (Zhao et al., 2018). Also, the data include the bond lengths and angles of [Ln_2_(IMBA)_6_(dmp)_2_] (Ln=Eu^3+^, **1a**; Ln=Gd^3+^, **1b**; Ln=Tb^3+^, **1c**).

**Specifications table**

**Table:** 

Subject area	*Chemistry*
More specific subject area	*FT-IR, PXRD, structural bond lengths, angles data of lanthanide complexes*
Type of data	*Table, figure*
How data was acquired	*Crystallography open data base and crystallographic tool – Diamond: Crystallographic Information File Code:* 1852307–1852309*.cif*
Data format	*Analyzed*
Experimental factors	*Single crystal X-ray diffraction (SCXRD) data was collected on a Bruker SMART 1000 CCD at 298(2) K, with Mo-Ka radiation (0.71073Å) at room temperature. The structure was refined by full-matrix least-squares methods with SHELXL-97 module. These single crystals are isostructural and they crystallize in triclinic space group P-1 (no. 2).*
Experimental features	*Block or needle-like colorless single crystal.*
Data source location	*Jiangxi Normal University, Nanchang, China.*
Data accessibility	*The data are with this article.*
Related research article	*Li-Wen Ding, Zi-Qi Liu, Highly Luminescent Lanthanide Complexes Constructed by Bis-tridentate Ligand and as Sensor for Et*_*2*_*O, submitted.*

**Value of the data**

• This structure information would be valuable for FT-IR analysis of lanthanide complexes.
• This data would be worthy for further investigation of the PXRD properties.
• This data provide a new process to synthesize two ligands coordinated lanthanide complexes.

## 1 Data

**Fig. 1:**
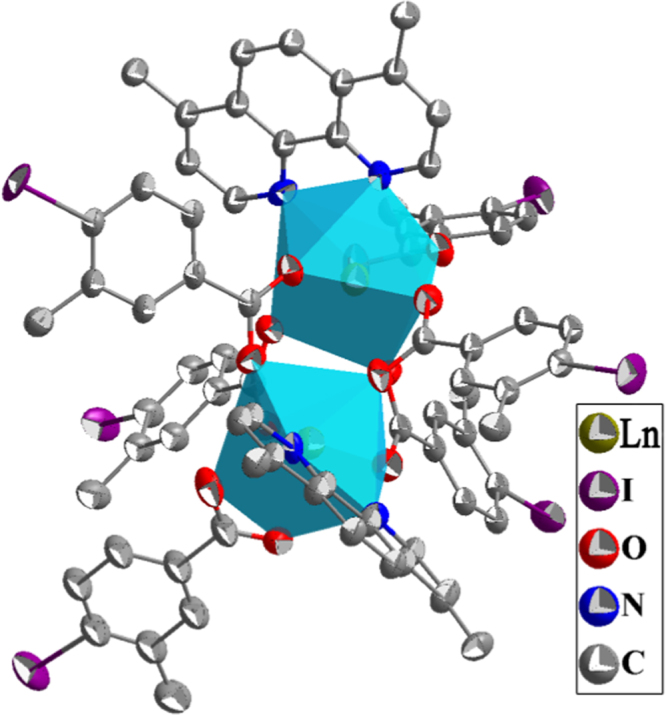
The dinuclear cluster structure.

**Fig. 2:**
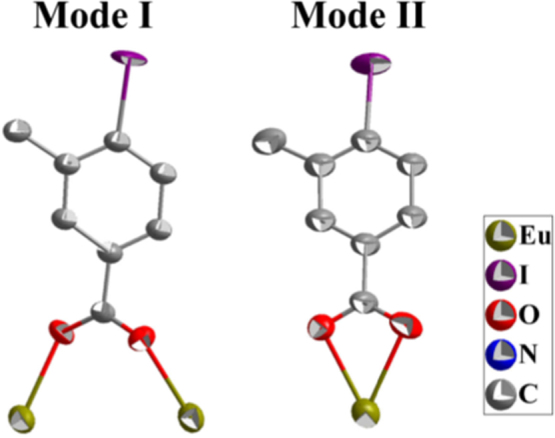
Coordination modes of the ligand IMBA in **1a**–**1c**.

**Fig. 3:**
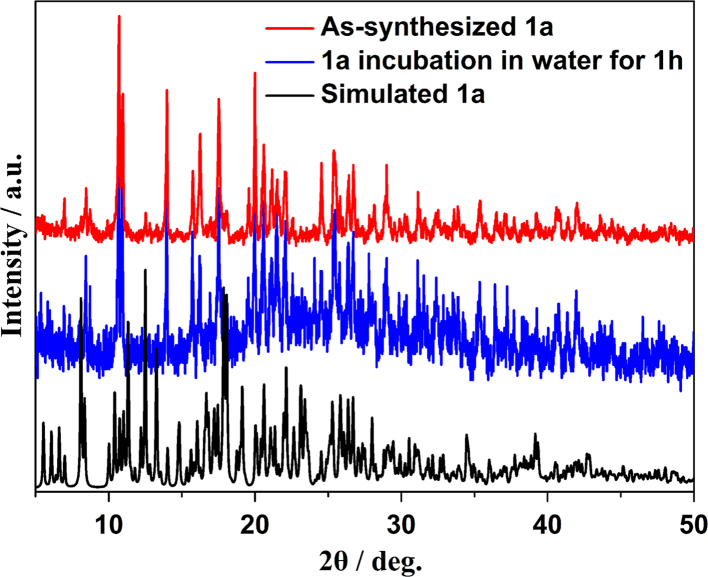
PXRD patterns comparison of simulated **1a**, as-synthesized **1a** and bulk sample **1a** immersed in water for 1 h, these peaks compete with each other very well, confirming **1a** is a stable sensor.

The single crystal structures of *[Ln*_*2*_*(IMBA)*_*6*_*(dmp)*_*2*_*] (Ln=Eu*^*3+*^*, **1a**; Ln=Gd*^*3+*^*, **1b**; Ln=Tb*^*3+*^*, **1c**)* are isostructural. They crystallize in triclinic space group P-1 (no. 2). These complexes are dinuclear cluster structures which contain two lanthanide ions (Ln^3+^, Ln1 and Ln2), six deprotonated IMBA and two dmp, forming an electroneutral unit (Fig. 1) [2]. In these complexes, IMBA has two coordination modes of bridge and chelation (Fig. 2). Ln–O and Ln–N bond lengths and bond angles are in line with the reported lanthanide complexes (Table 1, Table 2, Table 3) [3], [4], [5], [6], [7], [8]. PXRD of **1a** that incubation in aqueous solution for as long as the sensing time (1 h) was in line with the as-synthesized sample and calculated data, confirming that the sensor **1a** is a highly stable (Fig. 3) [9], [10], [11].

**Table 1:** Selected bond lengths and bond angles of **1a**.

Eu(1)–O(3)	2.319(6)	Eu(2)–O(6)	2.313(5)
Eu(1)–O(9)	2.350(5)	Eu(2)–O(8)	2.353(6)
Eu(1)–O(7)	2.367(6)	Eu(2)–O(10)	2.377(5)
Eu(1)–O(5)	2.376(6)	Eu(2)–O(4)	2.405(6)
Eu(1)–O(2)	2.432(6)	Eu(2)–O(12)	2.435(6)
Eu(1)–O(1)	2.439(6)	Eu(2)–O(11)	2.487(6)
Eu(1)–N(2)	2.600(7)	Eu(2)–N(4)	2.597(6)
Eu(1)–N(1)	2.629(7)	Eu(2)–N(3)	2.615(7)
Eu(1)–Eu(2)	4.2697(6)		
O(3)–Eu(1)–O(9)	74.2(2)	O(6)–Eu(2)–O(8)	78.3(2)
O(3)–Eu(1)–O(7)	116.2(2)	O(6)–Eu(2)–O(10)	117.1(2)
O(9)–Eu(1)–O(7)	77.1(2)	O(8)–Eu(2)–O(10)	77.4(2)
O(3)–Eu(1)–O(5)	76.7(2)	O(6)–Eu(2)–O(4)	80.1(2)
O(9)–Eu(1)–O(5)	133.8(2)	O(8)–Eu(2)–O(4)	134.0(2)
O(7)–Eu(1)–O(5)	84.7(2)	O(10)–Eu(2)–O(4)	77.2(2)
O(3)–Eu(1)–O(2)	83.6(2)	O(6)–Eu(2)–O(12)	153.0(2)
O(9)–Eu(1)–O(2)	76.5(2)	O(8)–Eu(2)–O(12)	128.6(2)
O(7)–Eu(1)–O(2)	140.7(2)	O(10)–Eu(2)–O(12)	75.9(2)
O(5)–Eu(1)–O(2)	134.3(2)	O(4)–Eu(2)–O(12)	80.2(2)
O(3)–Eu(1)–O(1)	83.9(2)	O(6)–Eu(2)–O(11)	149.9(2)
O(9)–Eu(1)–O(1)	127.5(2)	O(8)–Eu(2)–O(11)	78.2(2)
O(7)–Eu(1)–O(1)	153.3(2)	O(10)–Eu(2)–O(11)	75.3(2)
O(5)–Eu(1)–O(1)	83.3(2)	O(4)–Eu(2)–O(11)	129.9(2)
O(2)–Eu(1)–O(1)	53.7(2)	O(12)–Eu(2)–O(11)	53.0(2)
O(3)–Eu(1)–N(2)	147.7(2)	O(6)–Eu(2)–N(4)	82.8(2)
O(9)–Eu(1)–N(2)	77.8(2)	O(8)–Eu(2)–N(4)	137.9(2)
O(7)–Eu(1)–N(2)	72.0(2)	O(10)–Eu(2)–N(4)	144.2(2)
O(5)–Eu(1)–N(2)	135.4(2)	O(4)–Eu(2)–N(4)	77.7(2)
O(2)–Eu(1)–N(2)	74.3(2)	O(12)–Eu(2)–N(4)	75.1(2)
O(1)–Eu(1)–N(2)	100.9(2)	O(11)–Eu(2)–N(4)	102.6(2)
O(3)–Eu(1)–N(1)	148.8(2)	O(6)–Eu(2)–N(3)	80.1(2)
O(9)–Eu(1)–N(1)	136.5(2)	O(8)–Eu(2)–N(3)	77.1(2)
O(7)–Eu(1)–N(1)	75.2(2)	O(10)–Eu(2)–N(3)	145.1(2)
O(5)–Eu(1)–N(1)	75.6(2)	O(4)–Eu(2)–N(3)	137.5(2)
O(2)–Eu(1)–N(1)	106.0(2)	O(12)–Eu(2)–N(3)	102.6(2)
O(1)–Eu(1)–N(1)	78.8(2)	O(11)–Eu(2)–N(3)	76.5(2)
N(2)–Eu(1)–N(1)	62.1(2)	N(4)–Eu(2)–N(3)	62.7(2)

**Table 2:** Selected bond lengths and bond angles of **1b**.

Gd(1)–O(7)	2.306(5)	Gd(2)–O(10)	2.308(5)
Gd(1)–O(5)	2.341(5)	Gd(2)–O(4)	2.339(5)
Gd(1)–O(3)	2.367(5)	Gd(2)–O(6)	2.362(5)
Gd(1)–O(9)	2.380(5)	Gd(2)–O(8)	2.371(5)
Gd(1)–O(1)	2.437(5)	Gd(2)–O(11)	2.430(5)
Gd(1)–O(2)	2.488(5)	Gd(2)–O(12)	2.437(5)
Gd(1)–N(1)	2.588(6)	Gd(2)–N(4)	2.585(6)
Gd(1)–N(2)	2.591(6)	Gd(2)–N(3)	2.609(6)
O(7)–Gd(1)–O(5)	78.65(18)	O(10)–Gd(2)–O(4)	74.41(18)
O(7)–Gd(1)–O(3)	117.31(18)	O(10)–Gd(2)–O(6)	116.16(18)
O(5)–Gd(1)–O(3)	76.83(18)	O(4)–Gd(2)–O(6)	77.10(17)
O(7)–Gd(1)–O(9)	80.21(19)	O(10)–Gd(2)–O(8)	76.62(18)
O(5)–Gd(1)–O(9)	133.41(18)	O(4)–Gd(2)–O(8)	133.46(17)
O(3)–Gd(1)–O(9)	76.91(18)	O(6)–Gd(2)–O(8)	84.11(18)
O(7)–Gd(1)–O(1)	153.13(18)	O(10)–Gd(2)–O(11)	83.79(19)
O(5)–Gd(1)–O(1)	128.19(18)	O(4)–Gd(2)–O(11)	127.74(17)
O(3)–Gd(1)–O(1)	75.70(17)	O(6)–Gd(2)–O(11)	153.21(17)
O(9)–Gd(1)–O(1)	80.34(19)	O(8)–Gd(2)–O(11)	83.52(18)
O(7)–Gd(1)–O(2)	149.98(18)	O(10)–Gd(2)–O(12)	83.41(19)
O(5)–Gd(1)–O(2)	78.04(19)	O(4)–Gd(2)–O(12)	76.68(18)
O(3)–Gd(1)–O(2)	75.05(18)	O(6)–Gd(2)–O(12)	141.09(17)
O(9)–Gd(1)–O(2)	129.78(18)	O(8)–Gd(2)–O(12)	134.53(18)
O(1)–Gd(1)–O(2)	52.80(18)	O(11)–Gd(2)–O(12)	53.70(17)
O(7)–Gd(1)–N(1)	82.55(18)	O(10)–Gd(2)–N(4)	147.67(19)
O(5)–Gd(1)–N(1)	138.21(18)	O(4)–Gd(2)–N(4)	77.76(18)
O(3)–Gd(1)–N(1)	144.50(18)	O(6)–Gd(2)–N(4)	72.34(19)
O(9)–Gd(1)–N(1)	78.24(18)	O(8)–Gd(2)–N(4)	135.53(18)
O(1)–Gd(1)–N(1)	75.46(18)	O(11)–Gd(2)–N(4)	100.84(19)
O(2)–Gd(1)–N(1)	102.84(19)	O(12)–Gd(2)–N(4)	74.37(19)
O(7)–Gd(1)–N(2)	80.26(19)	O(10)–Gd(2)–N(3)	148.29(19)
O(5)–Gd(1)–N(2)	77.39(18)	O(4)–Gd(2)–N(3)	136.91(17)
O(3)–Gd(1)–N(2)	144.74(18)	O(6)–Gd(2)–N(3)	75.63(18)
O(9)–Gd(1)–N(2)	138.15(18)	O(8)–Gd(2)–N(3)	75.54(18)
O(1)–Gd(1)–N(2)	102.57(19)	O(11)–Gd(2)–N(3)	78.30(18)
O(2)–Gd(1)–N(2)	76.40(18)	O(12)–Gd(2)–N(3)	105.88(19)
N(1)–Gd(1)–N(2)	62.76(18)	N(4)–Gd(2)–N(3)	62.50(18)

**Table 3:** Selected bond lengths and bond angles of **1c**.

Tb(1)–O(10)	2.288(4)	Tb(2)–O(6)	2.295(4)
Tb(1)–O(12)	2.322(4)	Tb(2)–O(3)	2.333(4)
Tb(1)–O(4)	2.344(4)	Tb(2)–O(11)	2.340(4)
Tb(1)–O(5)	2.360(4)	Tb(2)–O(9)	2.351(4)
Tb(1)–O(2)	2.422(4)	Tb(2)–O(7)	2.414(4)
Tb(1)–O(1)	2.478(4)	Tb(2)–O(8)	2.419(4)
Tb(1)–N(4)	2.563(5)	Tb(2)–N(1)	2.566(5)
Tb(1)–N(3)	2.579(5)	Tb(2)–N(2)	2.592(5)
O(10)–Tb(1)–O(12)	78.92(15)	O(6)–Tb(2)–O(3)	74.49(15)
O(10)–Tb(1)–O(4)	117.29(14)	O(6)–Tb(2)–O(11)	116.18(15)
O(12)–Tb(1)–O(4)	76.57(15)	O(3)–Tb(2)–O(11)	77.18(15)
O(10)–Tb(1)–O(5)	79.82(16)	O(6)–Tb(2)–O(9)	76.61(16)
O(12)–Tb(1)–O(5)	133.23(15)	O(3)–Tb(2)–O(9)	133.03(14)
O(4)–Tb(1)–O(5)	77.10(15)	O(11)–Tb(2)–O(9)	83.50(15)
O(10)–Tb(1)–O(2)	152.67(15)	O(6)–Tb(2)–O(7)	83.26(16)
O(12)–Tb(1)–O(2)	128.38(15)	O(3)–Tb(2)–O(7)	128.16(15)
O(4)–Tb(1)–O(2)	75.99(14)	O(11)–Tb(2)–O(7)	153.06(15)
O(5)–Tb(1)–O(2)	80.40(16)	O(9)–Tb(2)–O(7)	83.32(15)
O(10)–Tb(1)–O(1)	149.68(16)	O(6)–Tb(2)–O(8)	82.95(16)
O(12)–Tb(1)–O(1)	77.54(16)	O(3)–Tb(2)–O(8)	76.69(15)
O(4)–Tb(1)–O(1)	75.13(15)	O(11)–Tb(2)–O(8)	141.55(14)
O(5)–Tb(1)–O(1)	130.47(16)	O(9)–Tb(2)–O(8)	134.75(15)
O(2)–Tb(1)–O(1)	53.52(16)	O(7)–Tb(2)–O(8)	54.21(14)
O(10)–Tb(1)–N(4)	82.34(15)	O(6)–Tb(2)–N(1)	147.45(17)
O(12)–Tb(1)–N(4)	138.72(15)	O(3)–Tb(2)–N(1)	77.72(16)
O(4)–Tb(1)–N(4)	144.31(16)	O(11)–Tb(2)–N(1)	72.84(15)
O(5)–Tb(1)–N(4)	77.77(15)	O(9)–Tb(2)–N(1)	135.77(16)
O(2)–Tb(1)–N(4)	75.16(15)	O(7)–Tb(2)–N(1)	101.27(16)
O(1)–Tb(1)–N(4)	103.37(16)	O(8)–Tb(2)–N(1)	74.40(16)
O(10)–Tb(1)–N(3)	79.95(16)	O(6)–Tb(2)–N(2)	147.94(16)
O(12)–Tb(1)–N(3)	77.25(15)	O(3)–Tb(2)–N(2)	137.22(16)
O(4)–Tb(1)–N(3)	144.71(15)	O(11)–Tb(2)–N(2)	75.90(15)
O(5)–Tb(1)–N(3)	138.06(15)	O(9)–Tb(2)–N(2)	75.51(15)
O(2)–Tb(1)–N(3)	102.97(16)	O(7)–Tb(2)–N(2)	78.09(16)
O(1)–Tb(1)–N(3)	76.44(15)	O(8)–Tb(2)–N(2)	106.09(16)
N(4)–Tb(1)–N(3)	63.39(15)	N(1)–Tb(2)–N(2)	62.90(16)

### 1.1 FT-IR spectra of 1a–1c

**Fig. 4:**
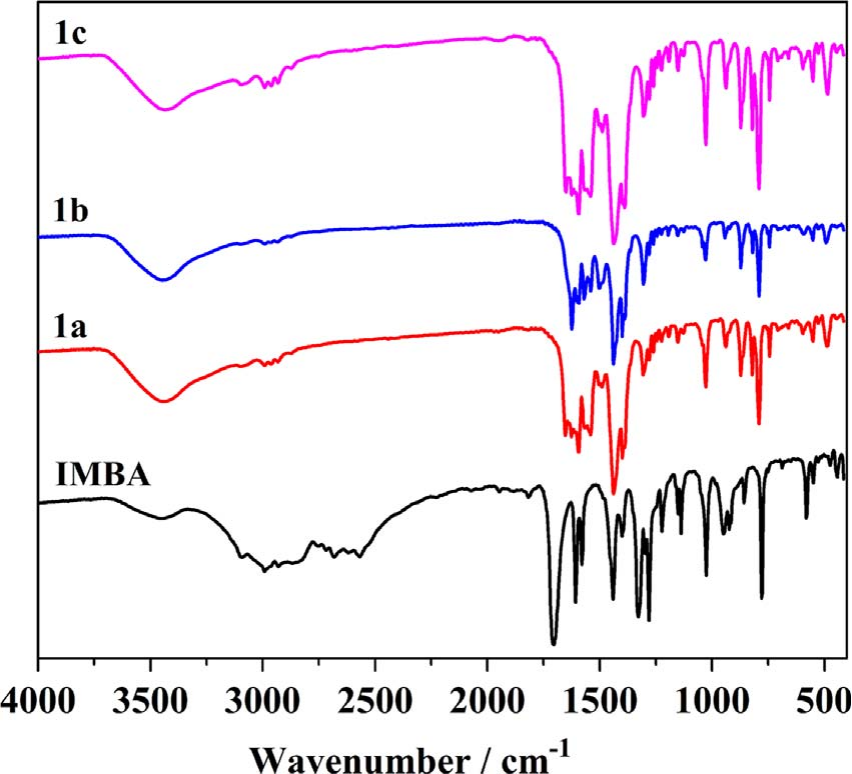
FT-IR spectra of the ligand IMBA and as-synthesized **1a**–**1c**.

FT-IR spectra of **1a**–**1c** (Fig. 4) are similar at 1710−1430 cm^−1^ and are assigned to C=O, C–C and C=C vibrations of the IMBA and dmp [12], [13]. The bands assignment at 1000–1300 cm^−1^ is difficult because of overlap [14], [15]. The coordination of carboxyl with Ln^3+^ is confirmed by the FT-IR: the stretching vibration of C=O decreases from 1647 to 1605 cm^−1^ and that of carboxyl O–H at 3400 cm^−1^ disappears.

[Eu_2_(IMBA)_6_(dmp)_2_]·(1a). Yield: 37% based on Eu^3+^. Anal. Calcd (%): C, 39.92; H, 2.645. Found (%): C, 40.13; H, 2.633. FT-IR (Fig. 4) (KBr pellet, cm^−1^): 3450 (m), 2947 (w), 1620 (s), 1584 (w), 1438 (s), 1403 (m), 1305 (m), 1193 (w), 1033 (m), 934 (w), 872 (m), 789 (s), 747 (w), 550 (w), 482 (w).

[Gd_2_(IMBA)_6_(dmp)_2_] (1b). Yield: 34% based on Gd^3+^. Anal. Calcd (%): C, 39.74; H, 2.633. Found (%): C, 39.61; H, 2.618. FT-IR (Fig. 4) (KBr pellet, cm^−1^): 3435 (m), 2995 (w), 1648 (w), 1592 (s), 1542 (m), 1495 (w), 1438 (s), 1305 (m), 1152 (w), 1033 (s), 943 (m), 872 (s), 789 (s), 739 (m), 600 (w), 550 (w), 488 (m).

[Tb_2_(IMBA)_6_(dmp)_2_] (1c). Yield: 35% based on Tb^3+^. Anal. Calcd (%): C, 39.68; H, 2.629. Found (%): C, 39.79; H, 2.637. FT-IR (Fig. 4) (KBr pellet, cm^−1^): 3443 (m), 2980 (w), 1627 (w), 1592 (s), 1542 (m), 1494 (w), 1430 (s), 1312 (w), 1152 (w), 1033 (s), 934 (w), 872 (m), 824 (m), 789 (s), 739 (w), 550 (w), 488(w).

## 2 Experimental design, materials, and methods

Lanthanide complexes **1a–1c** were synthesized with solvothermal method by heating a mixture of Ln(NO_3_)_3_·6H_2_O (Ln=Eu, Gd, Tb), dmp and IMBA at a molar ratio of 1:1:1.5 at 333 K for 120 h. The colorless block single crystals of **1a–1c** were collected by filtration, and mounted on a glass fiber [1].

Single crystal X-ray diffraction data were obtained on an instrument of Bruker SMART 1000 CCD, at wavelength of 0.71073 Å (Mo-Ka radiation) at 25 °C. The structures were refined by full-matrix least-squares methods with SHELXL-97 module. Phase purity of bulk samples were tested by PXRD, on a DMAX2200VPC diffractometer [7].
