# Supplementary material for: Structural data of lanthanide complex constructed by 4-iodo-3-methyl benzoic acid and 4,7-dimethyl-1,10-phenanthroline
Source: Data Brief. 2018 Sep 27;20:1928–34. doi: 10.1016/j.dib.2018.09.063 (PMC6171089; doi:10.1016/j.dib.2018.09.063)
Supplement: Supplementary file 1 — Supplementary material. [file mmc1.docx]

**Conflicts of interest:**

I am here, represent all of the four authors in this manuscript declare that there is not any financial and personal relationship with other people or organizations.

Dr. Yongli Zhao

Jiangxi Normal University

-----------------------------------------------------
